# Cell Membrane-Modified Lipid Nanoparticle Enhanced Glioblastoma Immunotherapy via Metabolism Reprogramming and Pyroptosis Induction

**DOI:** 10.3390/pharmaceutics18070901

**Published:** 2026-07-22

**Authors:** Pengxuan Zhao, Yu Tian, Weigang Yuan, Yang Bai, Yue Zhu, Liunuosi Wang, Ruoyi Wu, Fuchou Han, Ting Fan

**Affiliations:** 1 Hainan Provincial Key Laboratory of Research and Development on Tropical Herbs, Engineering Research Center of Tropical Medicine Innovation and Transformation of Ministry of Education, School of Pharmacy, Hainan Medical University, Haikou 571199, China; pengxuanzhao@muhn.edu.cn (P.Z.); 240115102@muhn.edu.cn (Y.B.); 2936354395@muhn.edu.cn (Y.Z.); 230901225wang@muhn.edu.cn (L.W.); wu.r.y@muhn.edu.cn (R.W.); hanfc@muhn.edu.cn (F.H.); 2 Department of Pharmacy, Union Hospital, Tongji Medical College, Huazhong University of Science and Technology, Wuhan 430022, China; 3 Key Laboratory of Combinatorial Biosynthesis and Drug Discovery (Ministry of Education), School of Pharmaceutical Sciences, Wuhan University, Wuhan 430071, China; tianyu@whu.edu.cn; 4 Department of Clinical Laboratory, Xianning Central Hospital, The First Affiliated Hospital of Hubei University of Science and Technology, Xianning 437000, China; 15629090416@hbust.edu.cn

**Keywords:** lipid nanoparticles, cell membrane modification, metabolism reprogramming, pyroptosis induction, glioblastoma immunotherapy

## Abstract

**Background:** Glioblastoma (GBM) has emerged as a model of resistance to immunotherapy because of the immunosuppressive tumor microenvironment (TME), which is closely associated with tryptophan metabolism. Inhibiting the expression of indoleamine 2,3-dioxygenase-1 (IDO1, a key enzyme in tryptophan metabolism) is a promising strategy for improving the immunosuppressive TME. Meanwhile, Gasdermin B (GSDMB)-mediated pyroptosis is a newly identified mechanism for activating the immune response. **Methods:** We prepared a GBM cell membrane (CM)-modified lipid nanoparticle (CMLNP) to deliver CRISPR/Cas9 components and mRNA encoding the N-terminal domain of GSDMB (GSDMB^NT^ mRNA). **Results:** The CM modification endowed the LNP with a tumor homing/homotypic targeting effect. Then, CRISPR/Cas9 components realized the knockdown of the *IDO1* gene, thus remodeling the TME. GSDMB^NT^ mRNA triggers pyroptosis, thus eliciting an immune response. **Conclusions:** This system generated potent antitumor immunity and offered a novel strategy for GBM immunotherapy.

## 1. Introduction

As the most common malignant primary brain tumor, glioblastoma (GBM) exhibits high mortality, reflected in a median survival below 2 years and a 5-year survival rate of merely 5.8% [1]. The current treatment strategy primarily involves surgical resection followed by radiotherapy/chemotherapy [2]. Unfortunately, the infiltrative growth pattern of GBM cells within the brain leads to unclear tumor boundaries, so it is not practically possible to resect the entire tumor [3]. Furthermore, the presence of the blood–brain barrier (BBB) prevents most of the drugs from entering the brain [4]. Therefore, there is an urgent need to develop innovative strategies to improve GBM prognosis.

Immunotherapy, which promotes tumor regression by activating the immune response, is responsible for significant clinical advancements [5]. However, the clinical outcomes of GBM immunotherapy are still unsatisfactory, primarily due to the highly immunosuppressive tumor microenvironment (TME) of GBM. The TME is composed of GBM cells and stromal and infiltrating immune cells, such as macrophages, regulatory T cells (Tregs), and bone marrow-derived cells (BMDCs) [6,7,8]. In particular, immune metabolisms, including amino acid, glucose, and lipid metabolisms, play a key role in regulating the immune cell responses [9]. Among them, the metabolism of amino acids such as tryptophan (Trp) influences the differentiation of immune cells and tumor development [10,11]. Trp is catabolized via indoleamine-2,3-dioxygenase 1 (IDO1) into Kynurenine (Kyn), which then binds and activates the aryl hydrocarbon receptor (AHR), facilitating Treg accumulation and suppressing cytotoxic T-cell activity [12]. Thus, IDO1 is an endogenous immunosuppressive mediator, and could be a potential immunotherapy target for reprogramming the immunosuppressive TME of GBM by improving amino acid metabolism. Current methods for IDO1 inhibition mainly include small-molecule inhibitors and RNA interference technology. However, small-molecule inhibitors could not provide a durable response because of drug resistance, and RNA interference technology suffers from transient gene silencing [13,14,15]. Hence, there is an urgent need to develop alternative IDO1 inhibition approaches to remodel the TME.

Clustered regularly interspaced short palindromic repeats (CRISPR)/CRISPR-associated endonuclease protein 9 (Cas9) represent a revolutionary gene editing technology, which has markedly simplified the genome editing procedure by utilizing the single-guide RNA (sgRNA) for target DNA recognition and the Cas9 nuclease to induce sequence-specific double-strand breaks (DSBs) [16,17,18]. CRISPR/Cas9 technology is expected to achieve the permanent inhibition of *IDO1*, thus reprogramming the amino acid metabolism and remodeling the highly immunosuppressive TME of GBM. In addition, immunogenic cell death (ICD) is essential to initiate the immune response [19]. Immunogenic apoptosis is the most commonly used type of ICD, which releases damage-associated molecular patterns (DAMPs) and tumor-associated antigens (TAAs) for activating the immune response [20]. However, the immunogenicity of released DAMPs and TAAs would be dramatically reduced in the apoptosis process because apoptosis is usually accompanied by intracellular oxidation and proteolysis, ultimately leading to a weak immune response [21,22]. In contrast, pyroptosis, induced by the proteolytic cleavage of gasdermin (GSDM) family proteins, is a form of inflammatory programmed cell death [23]. The pyroptosis process involves cell swelling and plasma membrane pore formation, resulting in the rapid release of DAMPs and pro-inflammatory cytokines, thereby activating a stronger immune response [24]. The GSDM is generally self-inhibited via the intramolecular interaction between their N-terminal and C-terminal domains [25]. Cleavage of the linker region induces the N-terminal domain to form oligomers. Oligomers move to the plasma membrane and subsequently create pores in the cell membrane, leading to rapid cell membrane rupture and DAMPs release [26,27]. Among the GSDM family, the N-terminal domain of the GSDMB protein could induce more pronounced pyroptosis [28]. Hence, we hypothesized that pyroptosis induction through the direct delivery of messenger RNA (mRNA) encoding the GSDMB N-terminal domain (GSDMB^NT^ mRNA) could be an effective method of initiating the immune response. Furthermore, GSDMB^NT^ mRNA in combination with CRISPR/Cas9 components (Cas9 mRNA and *IDO1* sgRNA) might induce a stronger immunotherapy effect for GBM.

However, the large size of mRNA and sgRNA is an obstacle for delivery [29,30]. Lipid nanoparticle (LNP) delivery is a clinically approved delivery system, which is suitable for delivering large-sized RNA [31]. Delivering the LNP across the BBB and into GBM cells poses an additional challenge. Recently, biomimetic nanoparticles based on cell membrane (CM) modification have gradually emerged [32,33]. Once coated with GBM CM, these nanoparticles could realize GBM cell targeting by tumor homing and homotypic targeting due to the intact copy of surface antigens from CM [34,35].

**Figure 1:**
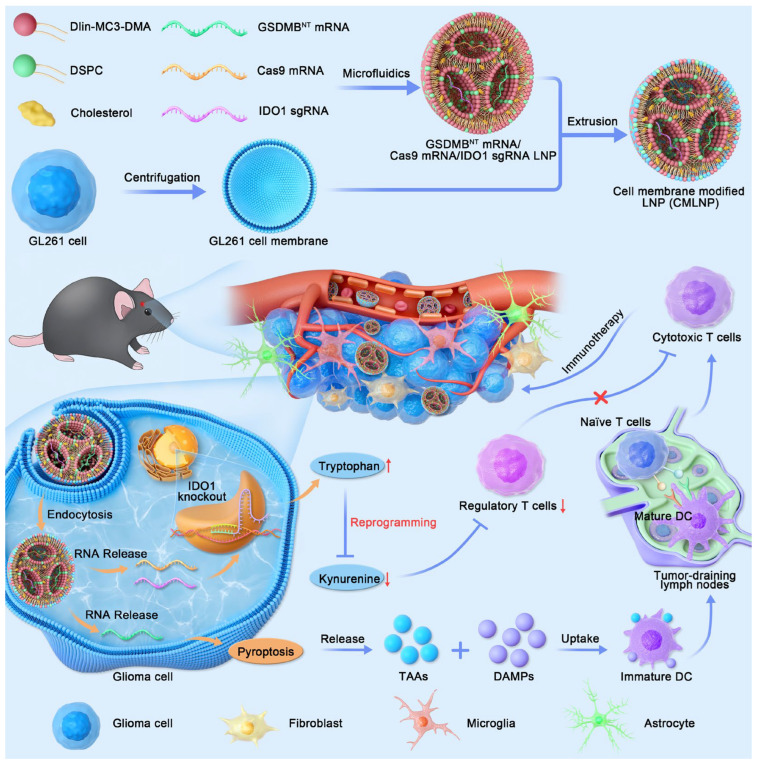
Schematic of the GSDMB^NT^/Cas9/sgIDO1-CMLNP for the BBB penetration, TME reprogramming, and pyroptosis induction in GBM.

Here, we report a GBM CM-coated LNP (CMLNP) for GSDMB^NT^ mRNA, Cas9 mRNA, and *IDO1* sgRNA delivery, named GSDMB^NT^/Cas9/sgIDO1-CMLNP (Figure 1). After intravenous injection, the GSDMB^NT^/Cas9/sgIDO1-CMLNP crossed the BBB and entered the GBM cell via CM coating. Then, GSDMB^NT^ mRNA was translated to the N-terminal domain of GSDMB, which induced pyroptosis. Subsequently, pyroptosis-released DAMPs and TAAs were taken up and processed through dendritic cells (DCs). After activation, mature DCs presented antigens to T cells and triggered antitumor immunity. Simultaneously, Cas9/sgIDO1 was transported to the nucleus for *IDO1* knockdown, reprogramming the amino acid metabolism and decreasing Tregs in the TME. Based on the results, we demonstrate that GSDMB^NT^/Cas9/sgIDO1-CMLNP can cross the BBB and target GBM cells, subsequently both remodeling the TME via CRISPR/Cas9-induced *IDO1* knockdown and initiating the immune response via pyroptosis; thus, strong immunotherapy can be achieved.

## 2. Materials and Methods

### 2.1. Materials

Dulbecco’s modified Eagle’s high-glucose medium (DMEM) and fetal bovine serum (FBS) were purchased from Wuhan Procell Biotechnology Co., Ltd (Wuhan, China). GL261 cells, as well as their luciferase transgenic counterparts (GL261-Luc), were kindly provided by Dr. Minjie Wang (Union Hospital, Huazhong University of Science and Technology). mRNA encoding firefly luciferase (Luc) was acquired from Vazyme Biotech Co., Ltd. (Nanjing, China). GSDMB N-terminal domain (GSDMB^NT^) mRNA, Cas9 mRNA, and Cy5-labeled mRNA (Cy5-mRNA) were purchased from Absin (Shanghai, China). Single-guide RNA (sgRNA), including *Luc* sgRNA (sgLuc) and *IDO1* sgRNA (sgIDO1), were purchased from Integrated DNA Technologies (IDT). D-Luciferin potassium salt was purchased from Yeasen Biotechnology (Shanghai, China).

### 2.2. Isolation of GL261 Cell Membrane (CM)

GL261 CM was prepared as in previous studies [36,37]. After collection, GL261 cells were rinsed with PBS three times and incubated in hypotonic lysing buffer overnight at 4 °C. The cell solution was then subjected to five repeated freeze–thaw cycles, each consisting of freezing at −80 °C and warming at 37 °C. Subsequent centrifugation at 700× *g* for 5 min at 4 °C yielded a supernatant, which was then centrifuged again at 14,000× *g* for 30 min at 4 °C to harvest the GL261 CM. The CM was reconstituted in ultrapure water and preserved at −80 °C. The protein content of the CM was quantified via bicinchoninic acid (BCA) assay.

### 2.3. Preparation and Characterization of CMLNP

The lipid nanoparticle (LNP) was prepared according to the previous studies [38,39]. DLin-MC3-DMA, cholesterol, and DSPC (molar ratio of 50/38.5/10) were mixed to form ethanol phase. GSDMB^NT^ mRNA/Cas9 mRNA/sgRNA (1:1:1 molar ratio) or Luc mRNA and citrate buffer were used to form aqueous phase. Subsequently, the two phases were mixed via microfluidics to prepare the mRNA-loaded LNP.

The GL261 CM-coated LNP (CMLNP) was prepared via a co-extrusion method. Briefly, the LNP was mixed with CM solution (1:1, mass ratio of mRNA and protein), and the mixtures were passed through a 200 nm polycarbonate membrane 15 times using a mini extruder (Avanti Polar Lipids, Alabaster, AL, USA) for co-extrusion. The B16F10 CM-coated LNP (CMLNP) was prepared by the same method. Transmission electron microscopy (TEM) was employed to examine the NP morphologies, while the RNA encapsulation efficiency was measured via the Quant-iT RiboGreen RNA assay.

RIPA lysis buffer was used to extract proteins from GL261 CM and CMLNP at 4 °C for 10 min. The cleared lysates were resolved by SDS-PAGE and electro-transferred onto PVDF membranes (Millipore, Burlington, MA, USA). Following a 1 h blockade in 5% non-fat milk, the membranes were incubated with rabbit monoclonal antibodies targeting EpCAM (Abcam (Cambridge, UK), ab223582, EPR20532-225) and Na^+^/K^+^ ATPase (Abcam, ab76020, EP1845Y) at 4 °C overnight. After washing, the membranes were reacted with anti-rabbit IgG secondary antibody (1:10,000) for 1 h at ambient temperature. The protein bands were developed by ECL (Amersham Imager 680RGB, GE, Tokyo, Japan), and Na^+^/K^+^ ATPase was used as a loading control.

### 2.4. Bone Marrow-Derived Dendritic Cells (BMDCs) Maturation

BMDCs were isolated from C57BL/6 mice as previously described [40]. The BMDCs were co-incubated with GSDMB^NT^ mRNA CMLNP-treated GL261 cells for 24 h. Then, BMDCs were harvested and stained with anti-CD11c antibody and anti-CD86 antibody. Ultimately, the cells were analyzed by FACS.

### 2.5. Animals and Mouse Tumor Model

Female C57BL/6 mice (5–6 weeks) were obtained from Beijing Vital River Laboratory Animal Technology Co., Ltd. (Beijing, China). All animal procedures received approval from the Committee on Ethical Animal Experimentation at Hainan Medical University. For orthotopic intracranial glioma modeling, C57BL/6 recipients were first anesthetized with isoflurane and secured in a stereotaxic frame. A suspension containing 1.0 × 10^5^ GL261 or GL261-Luc cells (in 5 µL volume) was then stereotactically injected into the right striatum at coordinates 1 mm anterior and 2 mm lateral to bregma, with a depth of 3.5 mm. Finally, the incision was closed using surgical glue.

### 2.6. In Vivo Biodistribution and Safety Study

For the biodistribution assessment, an orthotopic GL261 glioblastoma mouse model was employed. Luciferase-encoding mRNA (Luc mRNA) was encapsulated in CMLNPs. Either Luc mRNA-LNP or Luc mRNA-CMLNP were administered via intravenous injection at a mRNA dose of 0.25 mg/kg. Six hours later, the D-luciferin substrate (30 mg/mL) was given intraperitoneally. After an 8 min interval, bioluminescence imaging was performed using an IVIS Spectrum system (PerkinElmer, Waltham, MA, USA).

To examine acute toxicity, healthy C57BL/6 mice were injected intravenously with PBS, Cas9/sgIDO1-CMLNP, GSDMB^NT^ mRNA-CMLNP, or GSDMB^NT^/Cas9/sgIDO1-CMLNP, all at the same mRNA dosage (0.25 mg/kg). Subsequently, blood samples were collected, and the levels of alanine transaminase (ALT), aspartate transaminase (AST), and blood urea nitrogen (BUN) were measured with respective assay kits. Histopathological evaluation of major organs (heart, liver, spleen, lung, and kidney) was carried out using hematoxylin and eosin (H&E) staining, and the stained sections were finally examined under an optical microscope.

### 2.7. In Vivo Antitumor Experiment

The GL261-Luc mice were randomly divided into 4 groups (*n* = 5 each) and intravenously injected on days 4, 6, 8, and 10 post-implantation with PBS, Cas9/sgIDO1-CMLNP, GSDMB^NT^ mRNA-CMLNP, or GSDMB^NT^/Cas9/sgIDO1-CMLNP, each dose containing 0.5 mg/kg of mRNA. Therapeutic efficacy was assessed via bioluminescence imaging using the IVIS Spectrum system on days 5, 15, and 20 after tumor inoculation, while body weights were monitored routinely throughout the experiment.

After isolation, brains were homogenized in tissue protein extraction reagent supplemented with 1% proteinase and phosphatase inhibitors. The homogenates were gently rotated at 4 °C for 30 min then centrifuged to eliminate debris. The supernatants were used for Trp, Kyn, TNF-α, and IFN-γ ELISA analysis.

For immunofluorescence, isolated brains were initially fixed overnight in 4% paraformaldehyde, sequentially dehydrated in 15% and 30% sucrose solutions, each for 24 h, and finally stained with IDO1, HMGB1, CD4, and CD8 antibodies. Images were acquired via a fluorescence microscope (Olympus SZX12, Tokyo, Japan).

All other materials and methods are described in detail in the supporting information.

## 3. Results and Discussion

### 3.1. The Preparation and Characterization of Nanoparticles

**Figure 2:**
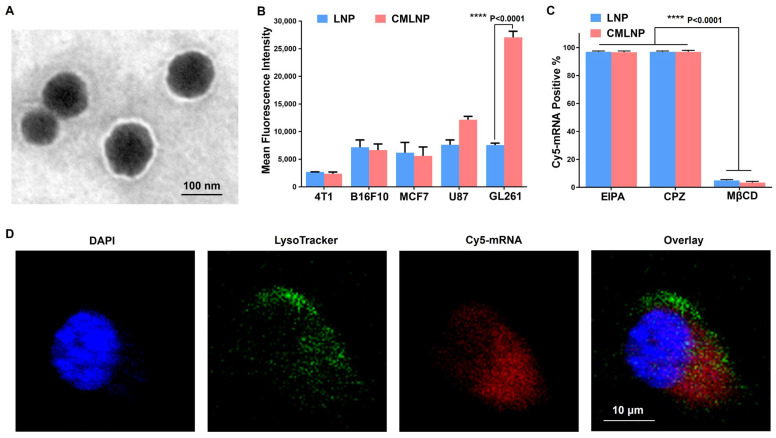
Characterization and cellular uptake of CMLNP: (**A**) TEM image of CMLNP. (**B**) Homotypic targeting through fluorescence measurement of the LNP or CMLNP incubated with different cell lines. (**C**) Cellular uptake in the presence of endocytic inhibitors, EIPA, CPZ, and MβCD, which inhibit macropinocytosis, clathrin-, and caveolae-mediated endocytosis, respectively. (**D**) CMLNP-mediated lysosomal escape and cytoplasmic release of Cy5-mRNA. DAPI (blue), lysosome (green), Cy5-mRNA-CMLNP (red). Scale bar =10 μm. Data are expressed as mean ± SEM (*n* = 5). Statistical significance was analyzed via the two-tailed Student’s *t*-test. **** *p* < 0.0001.

The LNP was prepared as previously described [38,39]. Briefly, DLin-MC3-DMA, cholesterol, and DSPC (molar ratio of 50/38.5/10) were mixed to form ethanol phase. RNA and citrate buffer were used to form aqueous phase. Subsequently, the two phases were mixed together via microfluidics for preparing the RNA-loaded LNP. For the CMLNP preparation, the CM and LNP were mixed and co-extruded through a 200 nm polycarbonate membrane. As shown in Figure 2A, transmission electron microscopy (TEM) images proved that CMLNP possessed an extra outer layer compared with the LNP due to CM coating. In addition, the encapsulation efficiency of Cas9 mRNA and sgRNA in CMLNP was approximately 90%.

Subsequently, we performed Western blot analysis to further characterize the proteins present on free GBM CM and CMLNP [41]. The critical surface marker EpCAM, which is essential for homologous targeting, was detected in free GBM CM and CMLNP, but not in the LNP, demonstrating that CM had successfully coated the LNP (Figure S1A).

### 3.2. Cellular Uptake of CMLNP

To confirm that CMLNP could efficiently deliver RNA into brain tumor GL261 cells, Cy5-mRNA was used to test the cellular uptake among different cell lines, including 4T1, B16F10, MCF7, U87 and GL261 cells, via flow cytometry (Figure 2B). Comparing all cell lines, the mean fluorescence intensity of GL261 cells treated with CMLNP was remarkably stronger than that of those treated with the LNP, revealing that the GL261 CM coating promoted LNP uptake into GL261 cells via the homotypic targeting effect [42]. Moreover, the B16F10 CM-coated LNP did not increase the fluorescence intensity of GL261 cells, which proved that only the homologous membrane has this specific targeting effect (Figure S1B).

To further study the endocytic pathways and lysosomal escape of CMLNP, Cy5-mRNA was loaded into CMLNP. After incubation with methyl-beta-cyclodextrin (MβCD, an inhibitor of caveolae-dependent endocytosis), the endocytosis efficiencies of both the LNP and CMLNP dramatically decreased by about 95%, proving that caveolae-mediated endocytosis was the primary route for LNP and CMLNP uptake (Figure 2C). In addition, the confocal images showed that most of the red signal from Cy5-mRNA was not co-localized with the LysoTracker Green in lysosomes after 4 h of incubation, indicating the effective cytosolic delivery of mRNA via CMLNP (Figure 2D).

### 3.3. In Vitro Genome Editing and Immunogenic Pyroptosis Induction of CMLNP

**Figure 3:**
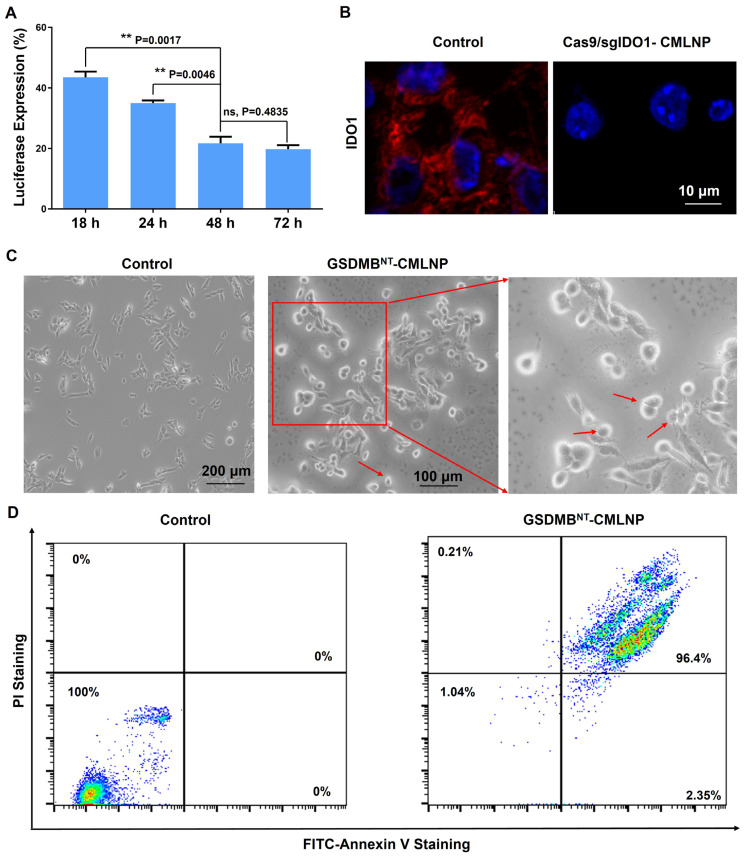
CMLNP induces genome editing and immunogenic pyroptosis. (**A**) In vitro gene-editing efficiency of Cas9/sgLuc-CMLNP. Data are expressed as mean ± SEM (*n* = 5). Statistical significance was analyzed by one-way ANOVA with Dunnett’s multiple comparison test. ** *p* < 0.01; ns: not significant. (**B**) Immunofluorescence staining for IDO1 expression with Cas9/sgIDO1-CMLNP treatment. DAPI was used to stain the nucleus of the cell (blue). (**C**) Cell morphologies of GSDMB^NT^-CMLNP-treated GL261 cells. The red arrows point to pyroptosis cells with cell swelling and membrane rupture characteristics. (**D**) Flow-cytometry analysis of cells positive for propidium iodide and annexin V.

Next, we tested the in vitro gene disruption efficiency of CMLNP encapsulating Cas9 mRNA and *luciferase* sgRNA (Cas9/sgLuc-CMLNP) by measuring the decrease in luciferase expression in GL261-Luc cells. As shown in Figure 3A, Cas9/sgLuc-CMLNP led to the lowest luciferase expression after 48 h and 72 h treatment, which reduced the luciferase expression to nearly 20%. To further verify the *IDO1* gene editing efficacy, CMLNP loading with Cas9 mRNA and *IDO1* sgRNA (Cas9/sgIDO1-CMLNP) was used to examine the *IDO1* degradation via immunofluorescence staining. The IDO1 protein expression was significantly decreased with Cas9/sgIDO1-CMLNP treatment, demonstrating that Cas9/sgIDO1 could realize the *IDO1* knockdown (Figure 3B). The above results proved that CMLNP could efficiently deliver RNA into GL261 cells and realize gene editing.

When treated with GSDMB^NT^ mRNA-loaded CMLNP (GSDMB^NT^-CMLNP), pyroptotic morphological features, including cytoplasmic swelling and membrane rupture, were detected (Figure 3C). Next, annexin V/propidium iodide (PI) staining was performed to test the lethal effect of GSDMB^NT^-CMLNP. The results in Figure 3D revealed that more than 90% of GL261 cells suffered programmed cell death after 24 h treatment with 250 ng/mL GSDMB^NT^ mRNA. Pyroptosis could release the DAMPs for ICD induction, thus triggering immune responses [43]. To confirm this, released DAMPs, including high mobility group box 1 (HMGB1) as well as adenosine triphosphate (ATP), were quantified in GL261 cells. GSDMB^NT^-CMLNP treatment significantly increased the extracellular secretion of HMGB1 and ATP compared with the empty CMLNP. As a result, the BMDCs maturation proportion of the GSDMB^NT^-CMLNP group rose to 46.4% (Figure S2). These results reveal that GSDMB^NT^ mRNA-mediated pyroptosis led to the DAMPs release, which induced the maturation of BMDCs.

### 3.4. Biodistribution and Safety Evaluation of CMLNP

To verify the brain tumor targeting of CMLNP in vivo, an orthotopic intracranial glioblastoma mouse model was constructed. Luc mRNA-LNP or Luc mRNA-CMLNP was intravenously injected (mRNA dose of 0.25 mg/kg). After 6 h, the bioluminescence signal was detected through an IVIS imaging system. Most of the luciferase signal translated by Luc mRNA-LNP was observed in the liver, while part of the luciferase signal could be detected in the brain site after treatment with Luc mRNA-CMLNP (Figure S3A). Moreover, Luc mRNA-CMLNP displayed nearly 3.4-fold higher bioluminescence signal intensity than Luc mRNA-LNP in the glioma area (Figure S3B). These results indicate that CM coating can assist LNP accumulation in the brain tumor site.

Before evaluating the therapeutic potential of CMLNP in vivo, we tested the toxicity of CMLNP in healthy C57BL/6 mice. An initial study of blood biochemistry parameters and HE staining proceeded after intravenous injection of PBS, Cas9/sgIDO1-CMLNP, GSDMB^NT^-CMLNP, or GSDMB^NT^/Cas9/sgIDO1-CMLNP. There were no significant differences in the liver enzyme levels (alanine transaminase, aspartate aminotransferase) and renal functions (blood urea nitrogen) across all the groups (Figure S4). Moreover, HE staining revealed no significant histopathological changes in the major organs (Figure S5). Although more thorough assessment of potential toxicity is needed for clinical translation, current results could prove that the prepared CMLNPs are not toxic at therapeutically relevant doses.

### 3.5. Anti-Glioma Activity of CMLNP

**Figure 4:**
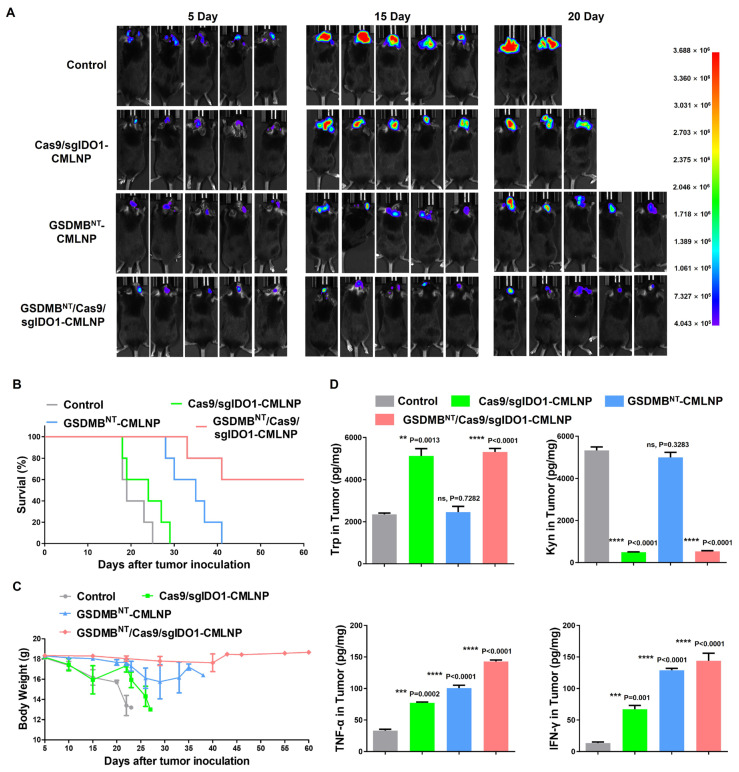
In vivo anti-glioma activity of GSDMB^NT^/Cas9/sgIDO1-CMLNP. (**A**) Bioluminescence images of GL261-Luc glioma-bearing mice with different treatments. (**B**) Survival curve for the mice. (**C**) Body weight for the mice. (**D**) ELISA of Trp, Kyn, TNF-α, and IFN-γ content in tumors after different treatments. Data are expressed as mean ± SEM (*n* = 5). Statistical significance was analyzed by one-way ANOVA with Dunnett’s multiple comparison test. ** *p* < 0.01, *** *p* < 0.001, **** *p* < 0.0001; ns: not significant.

To further test the immunotherapy effect of GSDMB^NT^/Cas9/sgIDO1-CMLNP, we investigated the antitumor efficacy in orthotopic GL261-Luc mouse model. In Figure 4A,B, the IVIS Spectrum images show that tumors grew rapidly in the Control and Cas9/sgIDO1-CMLNP groups; 3/5 (Control group) and 2/5 (Cas9/sgIDO1-CMLNP group) mice died within 20 days. The GSDMB^NT^-CMLNP group exhibited a moderate antitumor effect; the survival time of the GSDMB^NT^-CMLNP group was prolonged from 24 days in the Control group to 41 days. Moreover, the GSDMB^NT^/Cas9/sgIDO1-CMLNP group showed remarkable tumor inhibition, and 3/5 mice remained alive after 60 days. The body weights of the mice were significantly affected by the different therapies, which shows a trend similar to that of the survival rate (Figure 4C). To identify the mechanism of tumor growth inhibition by the GSDMB^NT^/Cas9/sgIDO1-CMLNP, the levels of Trp and Kyn, as well as those of the inflammatory cytokines, including tumor necrosis factor-alpha (TNF-α) and interferon-gamma (IFN-γ), in tumors were assayed via ELISA. As shown in Figure 4D, the Trp levels in the Cas9/sgIDO1-CMLNP and GSDMB^NT^/Cas9/sgIDO1-CMLNP groups were increased compared with the Control group, while the Kyn levels were decreased. In addition, the concentrations of TNF-α and IFN-γ were significantly elevated in the GSDMB^NT^/Cas9/sgIDO1-CMLNP group.

Altogether, these results demonstrate that the anti-glioma activity can be significantly amplified by GSDMB^NT^/Cas9/sgIDO1-CMLNP.

### 3.6. Tumor Microenvironment Remodeling of CMLNP

**Figure 5:**
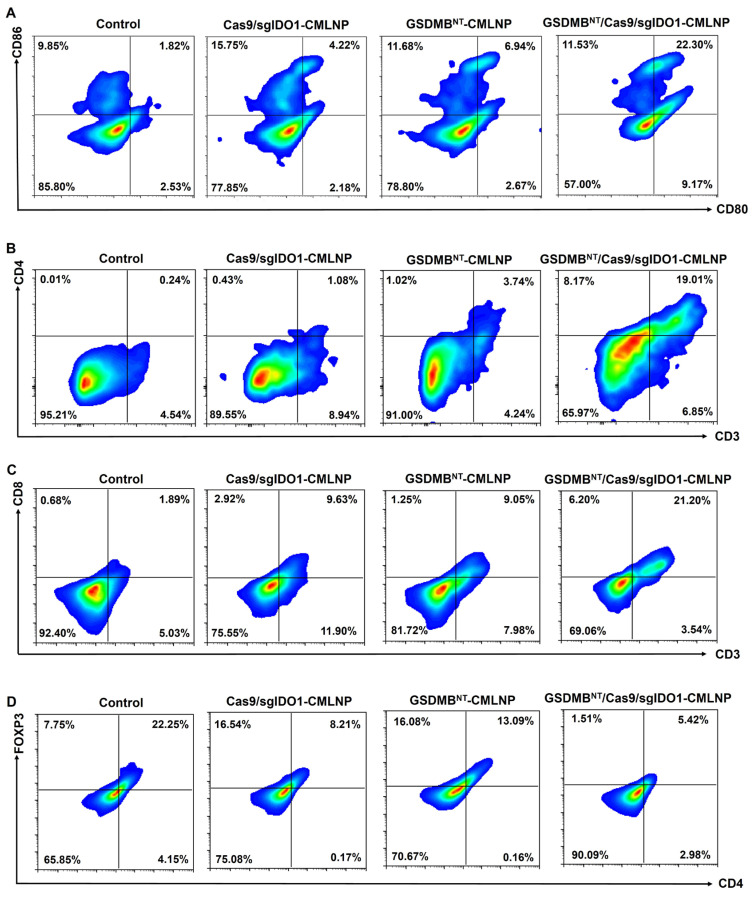
Remodeling of the immunosuppressive TME via GSDMB^NT^/Cas9/sgIDO1-CMLNP. Typical flow cytometric of mature DCs (**A**), CD4^+^ T cells (**B**), CD8^+^ T cells (**C**), and Tregs (**D**) in tumor tissues after treatment.

To identify how GSDMB^NT^/Cas9/sgIDO1-CMLNP remodel the tumor microenvironment, the immune cell populations in tumors were characterized via flow cytometry. Considering the important role of DCs in activating innate and adaptive immunity, we first detected the DC maturation in tumors. The results revealed that the proportions of mature DCs (CD11c^+^CD80^+^CD86^+^) in the Control, Cas9/sgIDO1-CMLNP, GSDMB^NT^-CMLNP, and GSDMB^NT^/Cas9/sgIDO1-CMLNP groups were 1.82%, 4.22%, 6.94%, and 22.30%, respectively (Figure 5A and Figure S7A). The DC maturation was significantly increase in the GSDMB^NT^/Cas9/sgIDO1-CMLNP group. Meanwhile, the population of CD4^+^ T cells and CD8^+^ T cells was also detected in the tumor tissue. CD4^+^ T cells and CD8^+^ T cells were recruited in tumors after GSDMB^NT^/Cas9/sgIDO1-CMLNP administration. Strikingly, compared to the Cas9/sgIDO1-CMLNP or GSDMB^NT^-CMLNP groups, the population of CD4^+^ T cells displayed a 17.6-fold or 5.1-fold increase, and CD8^+^ T cells displayed a 2.2-fold or 2.3-fold increase in the GSDMB^NT^/Cas9/sgIDO1-CMLNP group (Figure 5B,C and Figure S7B,C). To further verify the immunosuppression reduction via *IDO1* knockdown, the accumulation of Tregs in tumors was tested. The results showed that the proportion of Tregs in the Cas9/sgIDO1-CMLNP and GSDMB^NT^/Cas9/sgIDO1-CMLNP groups was significantly decreased (Figure 5D and Figure S7D). Furthermore, the alterations of M1- and M2-type macrophages in primary tumor cells were determined simultaneously. We observed that the tumor-suppressing M1-type macrophages in the GSDMB^NT^/Cas9/sgIDO1-CMLNP group were 3.0-fold higher than in the Control group, whereas the tumor-promoting M2-type macrophages were reduced by nearly 4.0-fold (Figures S6 and S8).

**Figure 6:**
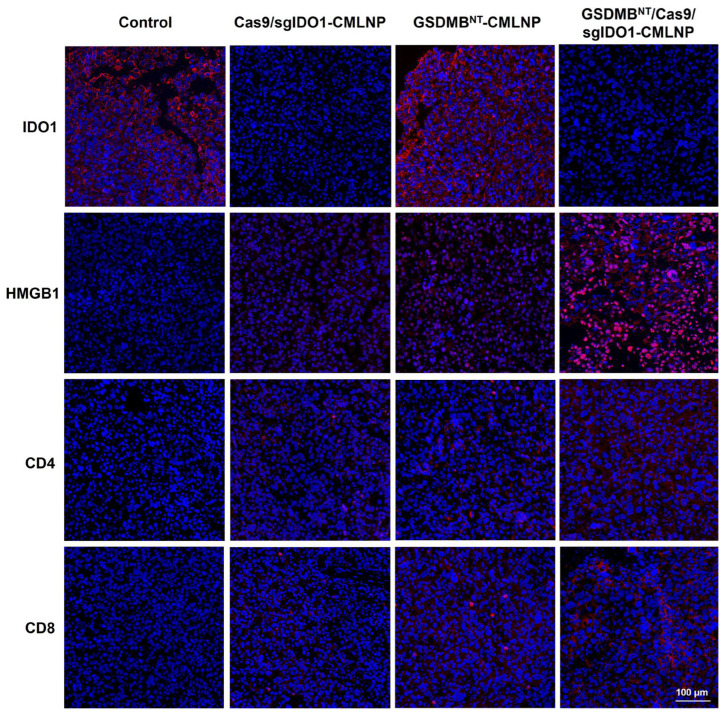
Immunofluorescence analysis of tumor tissues. Scale bar = 100 μm.

Immunofluorescence staining was also used to evaluate the expression of HMGB1 and IDO1 protein, as well as the proliferation of CD4^+^ T cells and CD8^+^ T cells, in tumor sections. As shown in Figure 6, the IDO1 protein level was significantly reduced in the Cas9/sgIDO1-CMLNP and GSDMB^NT^/Cas9/sgIDO1-CMLNP groups, indicating that the CRISPR/Cas9 system could realize *IDO1* knockdown. The expression of HMGB1 protein was significantly higher in the GSDMB^NT^/Cas9/sgIDO1-CMLNP group than in the other groups, suggesting that GSDMB^NT^/Cas9/sgIDO1-CMLNP could effectively induce ICD in vivo. The proliferation of CD4^+^ T cells and CD8^+^ T cells was significantly facilitated by the GSDMB^NT^/Cas9/sgIDO1-CMLNP treatment, suggesting the effective activation of immune system.

Collectively, these findings proved that GSDMB^NT^/Cas9/sgIDO1-CMLNP can effectively induce ICD, increase immunogenicity, and reverse the immunosuppressive microenvironment, thereby activating the anti-glioma immunity.

## 4. Conclusions

In summary, a cell membrane-modified LNP (CMLNP) was constructed for CRISPR/Cas9 components and GSDMB^NT^ mRNA delivery, thus realizing TME remodeling and pyroptosis induction for enhanced GBM immunotherapy. The cell membrane modification promoted the GBM targeting of the LNP. On reaching the GBM cells, the CRISPR/Cas9 components could knock down *IDO1*, reprogram amino acid metabolism, and decrease immunosuppressive cell infiltration. Meanwhile, GSDMB^NT^ mRNA could be translated to the N-terminal domain of GSDMB for pyroptosis induction, leading to DAMPs, TAAs release, and immune response activation. While our functional data collectively demonstrated the potent antitumor efficacy of the CMLNP platform, we acknowledge that the present study has certain limitations. First, DNA-level validation of genome editing was not performed. Second, we did not systematically evaluate potential CRISPR off-target effects. These issues should be addressed in future studies, for example, through whole-genome sequencing or targeted deep sequencing to assess the off-target effects.

## Data Availability

The original contributions presented in this study are included in the article/Supplementary Materials. Further inquiries can be directed to the corresponding authors.

## Supplementary Materials

The following supporting information can be downloaded at https://www.mdpi.com/article/10.3390/pharmaceutics18070901/s1: Figure S1: (A) Western blotting analysis of GL261 cell membrane special targeting-related protein. (B) Homotypic targeting through fluorescence measurement of the LNP, B16F10 CM-coated LNP, or GL261 CM-coated LNP incubated with GL261 cells. Figure S2: Release of ATP (A), HMGB1 (B), and BMDC maturation (C) after different treatments. Figure S3: (A) In vivo luminescence imaging results in mouse brain tumor model. (B) Quantitative bioluminescence signal intensity. Figure S4: Effect of different treatments on serum ALT, AST, and BUN levels. Figure S5: H&E staining of major organs. Figure S6: Typical flow cytometric of M1 macrophages (A) and M2 macrophages (B) in tumor tissues after treatment. Figures S7: Gating strategies for isolating mature DCs (A), CD4+ T cells (B), CD8+ T cells (C), and Tregs (D) from tumor tissue. Figures S8: Gating strategies for isolating M1 macrophages (A) and M2 macrophages (B) from tumor tissue.
