# Supplementary material for: Cell Membrane-Modified Lipid Nanoparticle Enhanced Glioblastoma Immunotherapy via Metabolism Reprogramming and Pyroptosis Induction
Source: Pharmaceutics. 2026 Jul 22;18(7):901. doi: 10.3390/pharmaceutics18070901 (PMC13414723; doi:10.3390/pharmaceutics18070901)
Supplement: Supplementary file 1 [file pharmaceutics-18-00901-s001.zip › pharmaceutics-4359318-supplementary.pdf]

Supplementary Materials

Materials

3-(4,5-dimethylthiazol-2-yl)-2,5-diphenyltetrazolium bromide (MTT), dimethyl sulfoxide (DMSO), and 4′,6-diamidino-2-phenylindole (DAPI) were purchased from Sigma-Aldrich Chemical Co (St. Louis, MO, USA). DLin-MC3-DMA, 1,2-dioctadecanoyl-sn-glycero-3-phophocholine (DSPC), and cholesterol were obtained from AVT Pharmaceutical Tech Co., Ltd (Shanghai, China). Cell culture dishes/plates, round coverslips, and centrifuge tubes were obtained from NEST Biotechnology Co. Ltd (Wuxi, China). Mouse high mobility group protein 1 (HMGB1), adenosine triphosphate (ATP), tryptophan (Trp), kynurenine (Kyn), TNF-α, and IFN-γ enzyme-linked immunosorbent assay (ELISA) kits were purchased from Elabscience Biotechnology Co. Ltd. Hypotonic lysing buffer, 5-(N-methyl-N-isopropyl) amiloride (EIPA), chlorpromazine (CPZ), and methyl-β-cyclodextrin (MβCD) were obtained from Beijing Solarbio Science & Technology Co., Ltd (Beijing, China).

Methods

Cellular uptake and mechanism analysis of CMLNP

4T1, B16F10, MCF7, U87, and GL261 cells were separately incubated with Cy5-mRNA-LNP or Cy5-mRNA-CMLNP (500 ng mRNA/well) for 8 h in 12-well plates. After washing and suspending in PBS, the average fluorescence was measured by a flow cytometer (FACS, LSRII, BD). Then, GL261 cells were separately incubated with Cy5-mRNA-LNP, Cy5-mRNA-B16F10-CMLNP, or Cy5-mRNA-GL261-CMLNP (500 ng mRNA/well) for 8 h in 12-well plates. After washing and suspending in PBS, the average fluorescence was measured by FACS.

GL261 cells were seeded in confocal dishes and incubated with Cy5-mRNA-CMLNP (250 ng mRNA/per dish) for 4 h. The cells were then washed with PBS 3 times and fixed in 4% para-formaldehyde for 15 min. Cells were stained with DAPI and LysoTracker Green. The fluorescence signals were analyzed by confocal laser scanning microscopy (CLSM, Olympus FV-IX81, Japan). To study endocytic pathways of CMLNP, a quantified cellular uptake assay was performed using a flow cytometer (FACS, LSRII, BD, CA, USA) in the presence of different endocytotic inhibitors, namely, EIPA, CPZ, and MβCD, which inhibit macropinocytosis-, clathrin-, and caveolae-mediated endocytosis, respectively.

Genome editing of CMLNP in vitro

First, the luciferase knockout assays were used to evaluate the genome editing efficiency in vitro. GL261-Luc cells were seeded in 96-well plates at 2×10^4 cells per well, cultured overnight, and then treated with Cas9 mRNA/sgLuc-loaded CMLNP (Cas9/sgLuc-CMLNP) at a concentration of 50 ng mRNA/well. After 18, 24, 48, or 72 h, 100 μL luciferase substrate was added to each well. After 5 min, the luminescence intensity was measured using the SpectraMax M5 microplate reader (Molecular Devices, LLC., Sunnyvale, CA, USA).

Then, sgIDO1 was used to further evaluate the genome editing via immunofluorescence staining. GL261 cells were seeded in 12-well plates containing 1.0 mL DMEM medium and

incubated with Cas9/sgLuc-CMLNP (50 ng mRNA/well). After 18 h, the cells were incubated with Alexa Fluor 647 anti-mouse IDO1 antibody (Biolegend, Cat#654003, Clone No. 2E2) for 2 h. Then, the cells were stained with DAPI for 20 min and captured via a fluorescence microscope (Olympus SZX12, Japan).

Pyroptosis evaluation of CMLNP in vitro

To examine the changes in cell morphology, GL261 cells were seeded in 12-well plates containing 1.0 mL DMEM medium and incubated with $GSDMB^{NT}$ mRNA-CMLNP (250 ng mRNA/well). After 24 h of treatment, a microscope was used to capture the changes in cell morphology. Then, annexin V-FITC and propidium iodide (PI) were added to the medium. After 15 min of incubation, a flow cytometer was used to test the number of annexin V-FITC- and PI-positive cells.

Assessment of extracellular DAMPs levels

To measure the extracellular high mobility group protein 1 (HMGB1) and adenosine triphosphate (ATP) level, GL261 cells were seeded in a 6-well plate (5.0×10^5 cells/well) and incubated with empty CMLNP or $GSDMB^{NT}$ mRNA-CMLNP (1 μg mRNA/well) for 10 h. The supernatant was collected and measured via the corresponding ELISA kit.

Flow cytometry for immune cells

$CD11c^+CD86^+CD80^+$ dendritic cells, $CD3^+CD4^+$ T cells, $CD3^+CD8^+$ T cells, $CD3^+CD4^+FOXP3^+$ T cells, $CD45^+CD11b^+F4/80^+CD86^+$ M1-like macrophages, and $CD45^+CD11b^+F4/80^+CD206^+$ M2-like macrophages in the brain were isolated and analyzed using flow cytometry.

The antibodies involved in the experiments include FITC anti-mouse CD11c antibody (Biolegend, Cat#117306, Clone No. N418), PE anti-mouse CD86 antibody (Biolegend, Cat#105007, Clone No.GL-1), APC anti-mouse CD80 antibody (Biolegend, Cat#104714, Clone No.16-10A1), FITC anti-mouse CD3 antibody (Biolegend, Cat#100203, Clone No.17A2), PE/Cyanine7 anti-mouse CD4 antibody (Biolegend, Cat#100421, Clone No.GK1.5), APC anti-mouse CD8a antibody (Biolegend, Cat#100712, Clone No.53-6.7), PE anti-mouse FOXP3 antibody (BD, Cat# 563101, Clone No.150D), APC anti-mouse CD45 antibody (Biolegend, Cat#103112, Clone No.30-F11), FITC anti-mouse CD11b antibody (Biolegend, Cat#101205, Clone No.M1/70), PerCP anti-mouse F4/80 antibody (Biolegend, Cat# 123126, Clone No.BM8), and PE/Cyanine7 anti-mouse CD206 antibody (Biolegend, Cat# 141720, Clone No.C068C2). The antibody concentration was 1:100 diluted with PBS.

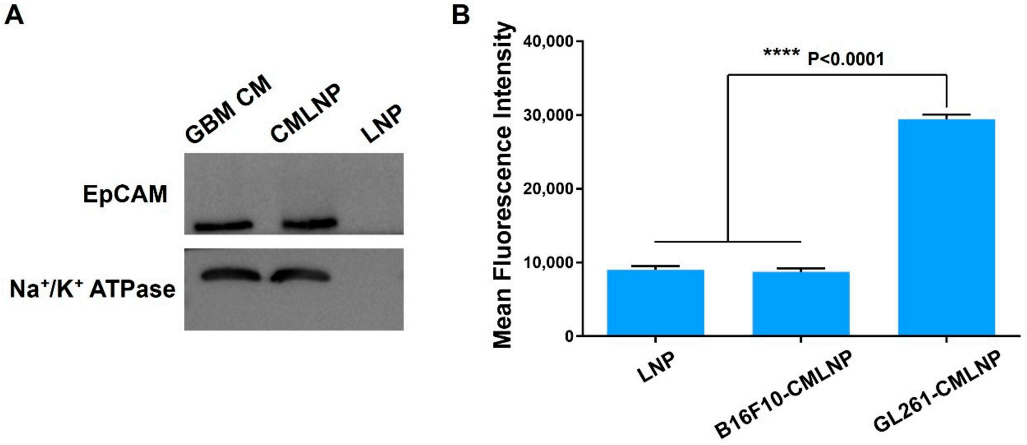

Figure S1. (**A**) Western blotting analysis of GL261 cell membrane special targeting-related protein. (**B**) Homotypic targeting through fluorescence measurement of the LNP, B16F10 CM-coated LNP, or GL261 CM-coated LNP incubated with GL261 cells. Data are expressed as mean ± SEM (*n* = 5). Statistical significance was analyzed by one-way ANOVA with Dunnett's multiple comparison test. ****P < 0.0001.

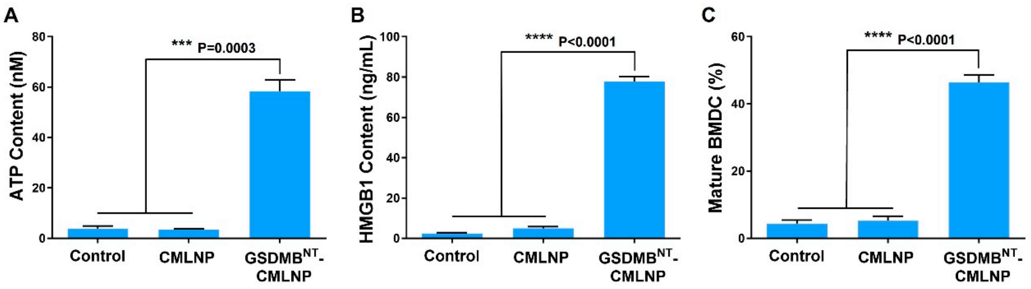

Figure S2. Release of ATP (**A**) and HMGB1 (**B**), and BMDC maturation (**C**) after different treatments. Data are expressed as mean ± SEM (*n* = 5). Statistical significance was analyzed by one-way ANOVA with Dunnett's multiple comparison test. ***P < 0.001, ****P < 0.0001.

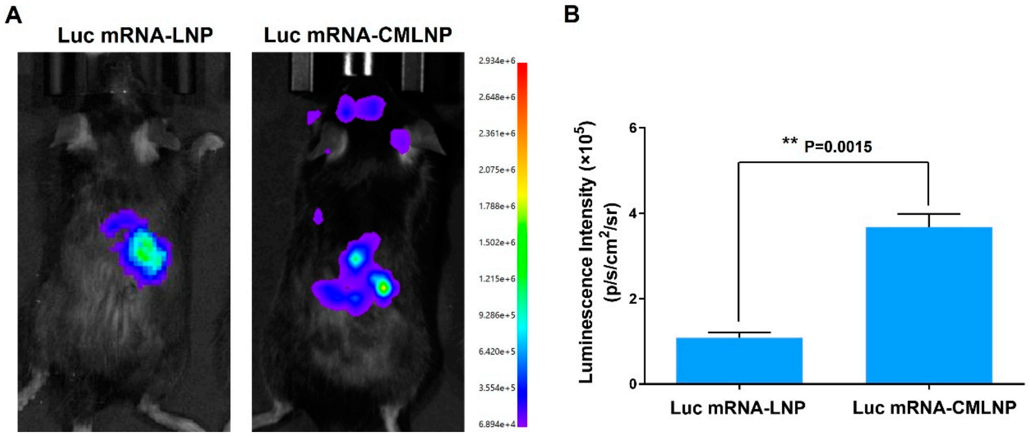

Figure S3. (**A**) In vivo luminescence imaging results in mouse brain tumor model. (**B**) Quantitative bioluminescence signal intensity. Data are expressed as mean ± SEM (n = 3). Statistical significance was analyzed by the two-tailed Student's t-test. **P < 0.01.

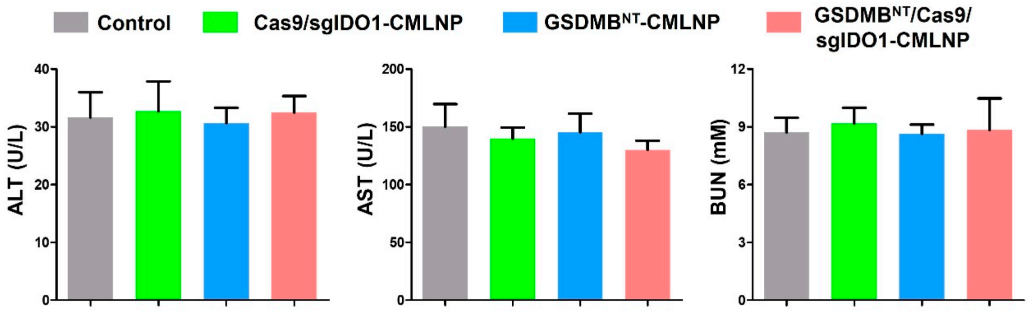

Figure S4. Effect of different treatments on serum ALT, AST, and BUN levels.

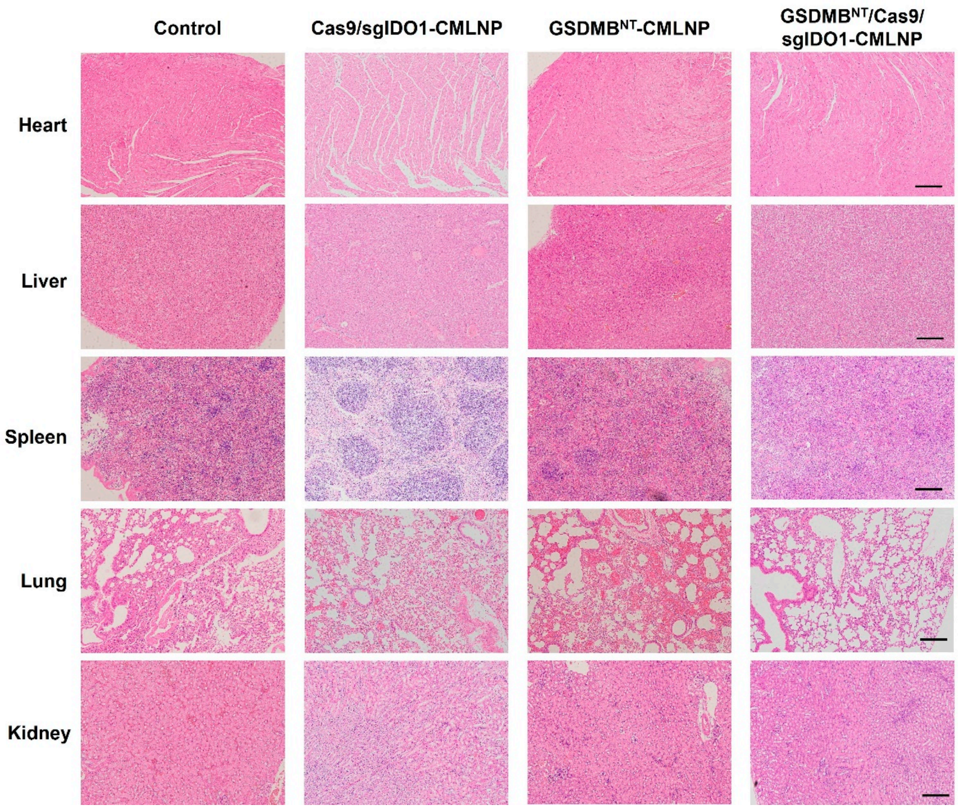

Figure S5. H&E staining of major organs after treatments. Scale bar: 100 μm.

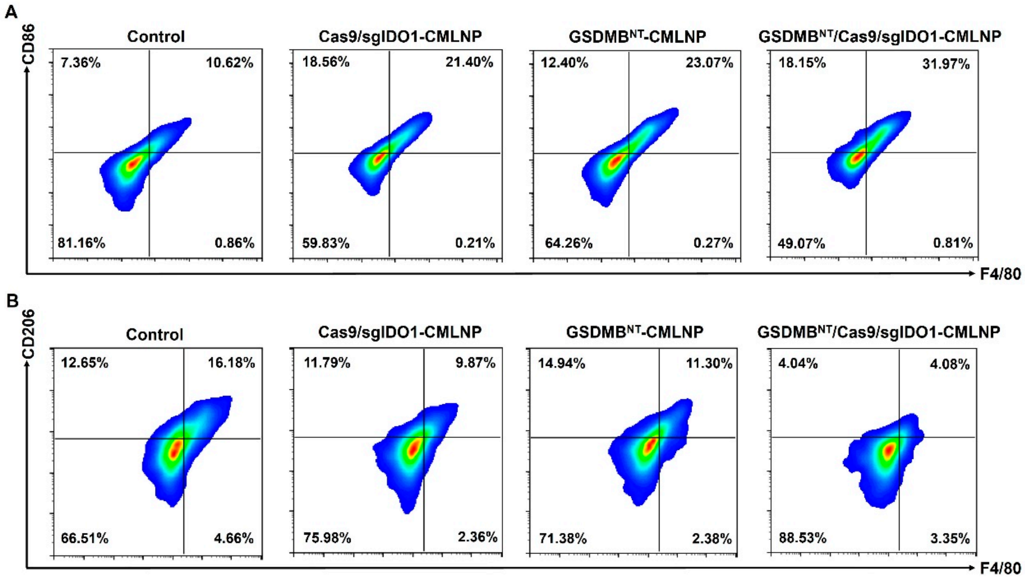

Figure S6. Typical flow cytometric of M1 macrophages (**A**) and M2 macrophages (**B**) in tumor tissues after treatment.

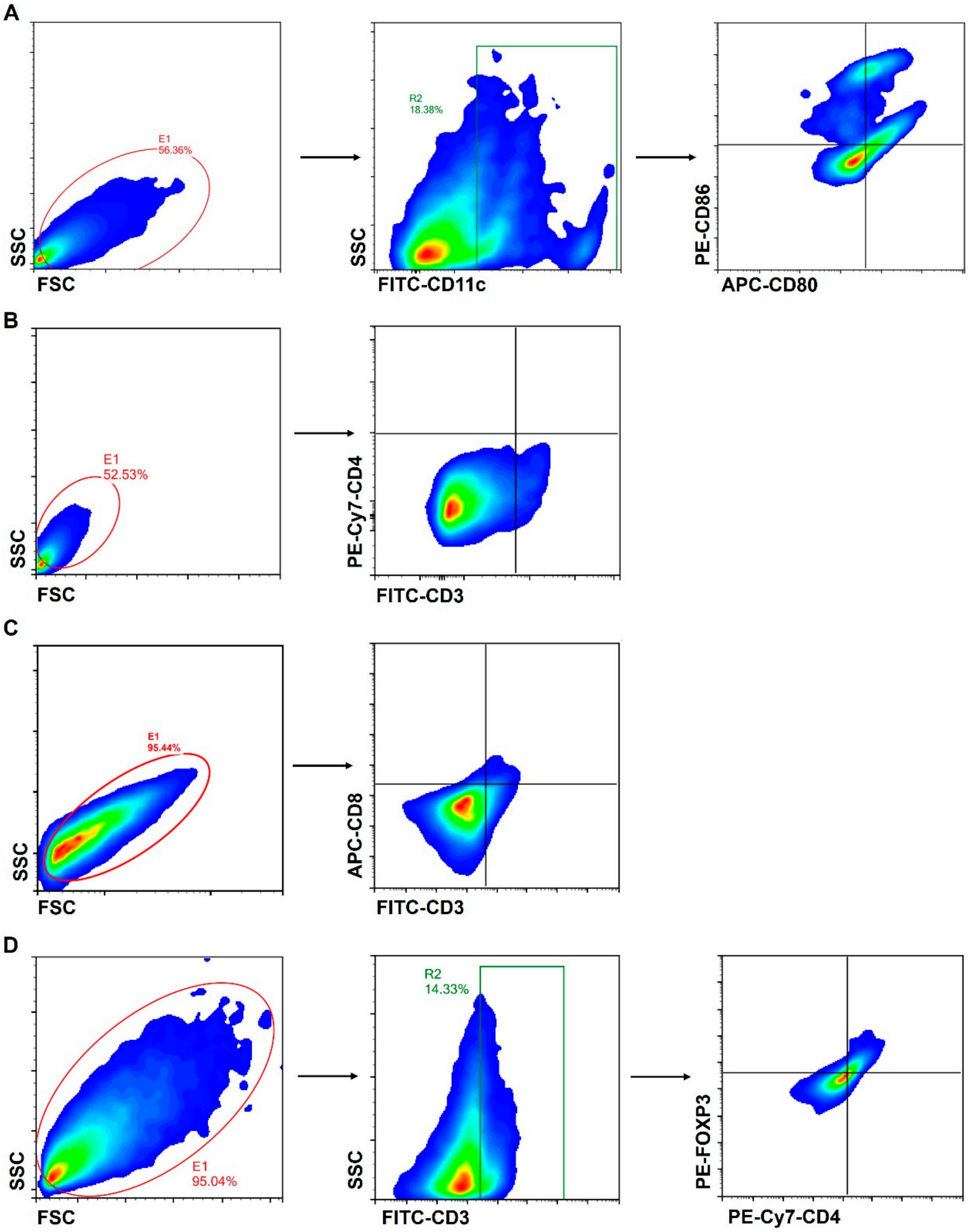

Figure S7. Gating strategies for isolating mature DCs (**A**), CD4⁺ T cells (**B**), CD8⁺ T cells (**C**), and Tregs (**D**) from tumor tissue.

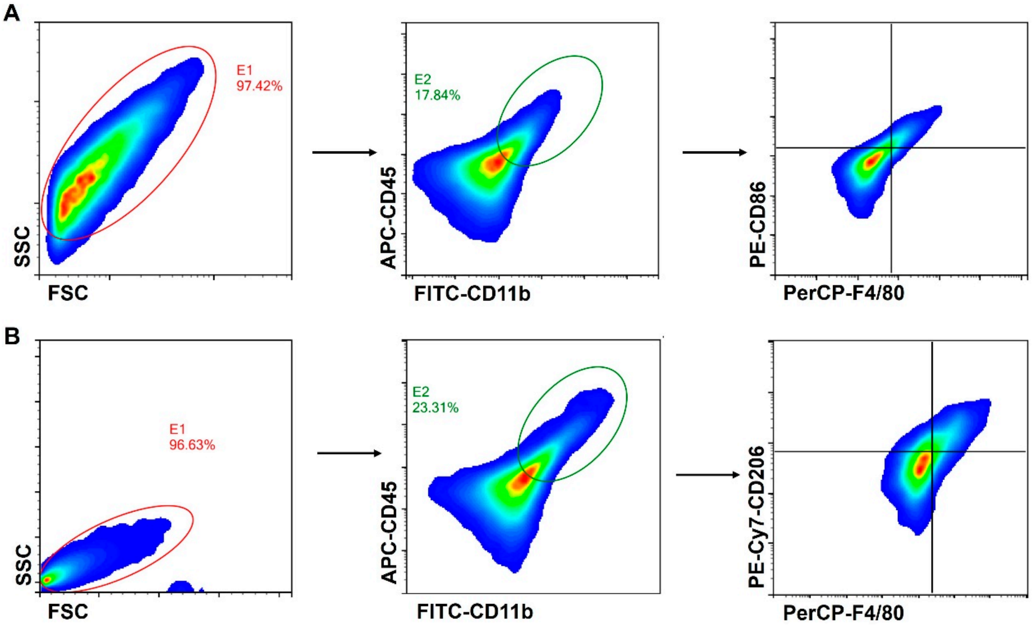

Figure S8. Gating strategies for isolating M1 macrophages (**A**) and M2 macrophages (**B**) from tumor tissue.
